# Tissue specificity of senescent cell accumulation during physiologic and accelerated aging of mice

**DOI:** 10.1111/acel.13094

**Published:** 2020-01-25

**Authors:** Matthew J. Yousefzadeh, Jing Zhao, Christina Bukata, Erin A. Wade, Sara J. McGowan, Luise A. Angelini, Michael P. Bank, Aditi U. Gurkar, Collin A. McGuckian, Mariah F. Calubag, Jonathan I. Kato, Christin E. Burd, Paul D. Robbins, Laura J. Niedernhofer

**Affiliations:** ^1^ Institute on the Biology of Aging and Metabolism University of Minnesota Minneapolis MN USA; ^2^ Department of Biochemistry, Molecular Biology and Biophysics University of Minnesota Minneapolis MN USA; ^3^ Department of Molecular Medicine Scripps Research Institute Jupiter FL USA; ^4^ Harriet L. Wilkes Honors College Florida Atlantic University Jupiter FL USA; ^5^ Charles E. Schmidt College of Medicine Florida Atlantic University Boca Raton FL USA; ^6^ Department of Medicine University of Pittsburgh Pittsburgh PA USA; ^7^ Departments of Molecular Genetics and Cancer Biology and Genetics The Ohio State University Columbus OH USA

**Keywords:** aging, cellular senescence, DNA repair, endogenous DNA damage, ERCC1‐XPF, progeria

## Abstract

Senescent cells accumulate with age in vertebrates and promote aging largely through their senescence‐associated secretory phenotype (SASP). Many types of stress induce senescence, including genotoxic stress. ERCC1‐XPF is a DNA repair endonuclease required for multiple DNA repair mechanisms that protect the nuclear genome. Humans or mice with reduced expression of this enzyme age rapidly due to increased levels of spontaneous, genotoxic stress. Here, we asked whether this corresponds to an increased level of senescent cells. *p16^Ink4a^* and *p21^Cip1^* mRNA were increased ~15‐fold in peripheral lymphocytes from 4‐ to 5‐month‐old *Ercc1* ^−/∆^ and 2.5‐year‐old wild‐type (WT) mice, suggesting that these animals exhibit a similar biological age. *p16^Ink4a^* and *p21^Cip1^* mRNA were elevated in 10 of 13 tissues analyzed from 4‐ to 5‐month‐old *Ercc1* ^−/∆^ mice, indicating where endogenous DNA damage drives senescence in vivo. Aged WT mice had similar increases of *p16^Ink4a^* and *p21^Cip1^* mRNA in the same 10 tissues as the mutant mice. Senescence‐associated β–galactosidase activity and p21*^Cip1^* protein also were increased in tissues of the progeroid and aged mice, while Lamin B1 mRNA and protein levels were diminished. In *Ercc1* ^−/Δ^ mice with a *p16^Ink4a^* luciferase reporter, bioluminescence rose steadily with age, particularly in lung, thymus, and pancreas. These data illustrate where senescence occurs with natural and accelerated aging in mice and the relative extent of senescence among tissues. Interestingly, senescence was greater in male mice until the end of life. The similarities between *Ercc1* ^−/∆^ and aged WT mice support the conclusion that the DNA repair‐deficient mice accurately model the age‐related accumulation of senescent cells, albeit six‐times faster.

## Graphical abstract

Senescent cells contribute to aging and its associated morbidities. Senescent cells accumulate in vertebrates with aging. Here, we survey where (in what tissues) senescence occurs with aging in mice, by measuring p16*^Ink4a^* and p21*^Cip1^* mRNA. A similar survey in *Ercc1* ^−/Δ^ mice illustrates where (in what tissues) senescence occurs in vivo as a consequence of endogenous DNA damage.

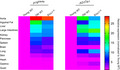

**Abbreviations**
ERCC1: excision repair cross‐complementation group 1
mRNA: messenger RNA
PBMC: peripheral blood mononuclear cell
qPCR: quantitative polymerase chain reaction
Rb: retinoblastoma protein
SASP: senescence‐associated secretory phenotype
SA‐βgal: senescence‐associated β‐galactosidase
WT: wild‐type
XPF: xeroderma pigmentosum complementation group F protein

## 1 INTRODUCTION

Cellular senescence is a programmed, largely irreversible arrest of cellular proliferation, sustained even in the presence of mitogenic stimuli (Campisi, 2013). Senescence can be induced by a variety of cell stressors, including telomere attrition, oncogene activation, and genotoxic or oxidative stress (Niedernhofer et al., 2018). Once a cell enters senescence, through the p53/p21^CIP1^, p16^INK4a^/pRB, or GATA4 pathways, it exhibits key changes in chromatin organization and gene expression (Campisi, 2013; Childs, Durik, Baker, & van Deursen, 2015; van Deursen, 2014; Kang et al., 2015) but remains metabolically active. Senescent cells can display a senescence‐associated secretory phenotype (SASP), comprised of pro‐inflammatory cytokines, chemokines, growth factors, and proteases that influence the surrounding environment through a paracrine mechanism (Coppe, Desprez, Krtolica, & Campisi, 2010; Tchkonia, Zhu, van Deursen, Campisi, & Kirkland, 2013). Senescent cell burden is well known to increase with age in vertebrates (Krishnamurthy et al., 2004; C. Wang et al., 2009). The accumulation of senescent cells in tissue negatively impacts tissue homeostasis and regeneration, likely via SASP, and thereby promotes aging (Baker et al., 2016, 2004; Tchkonia et al., 2013). Prior studies demonstrated that clearance of senescent cells in wild‐type (WT) and progeroid *Ercc1* ^−/∆^ or *BubR1* ^H/H^ mice improves health span, establishing that senescence drives aging and associated age‐related diseases (Baker et al., 2016; Chang et al., 2016; Zhu, Tchkonia, Fuhrmann‐Stroissnigg, et al., 2015; Zhu, Tchkonia, Pirtskhalava, et al., 2015). However, very little is known about when and where senescent cells accumulate with normal or accelerated aging.

Progeroid syndromes are genetic disorders characterized by the premature onset of aging‐like features in one or more organ systems. These syndromes, and murine models of them, have been informative in identifying factors that promote aging‐like physiological decline, for example, telomere shortening, expression of progerin, and DNA damage (Burtner & Kennedy, 2010; Gurkar & Niedernhofer, 2015; Hisama, Oshima, & Martin, 2016). However, there remains controversy as to whether progeroid syndromes reflect an acceleration of the normal aging process or a disease state (Miller, 2004; Warner & Sierra, 2003). The fact that cells and tissues from progeria patients and murine models of their diseases senesce prematurely suggests that at least certain progeroid syndromes represent the acceleration of normal aging processes (Burtner & Kennedy, 2010; Childs et al., 2015; Davis & Kipling, 2009; Gregg et al., 2012).

Reduced expression of the DNA repair endonuclease, ERCC1‐XPF, causes accelerated aging in humans and mice (XFE progeroid syndrome) (Niedernhofer et al., 2006). ERCC1‐XPF is a heterodimeric enzyme required for multiple DNA repair mechanisms (Gurkar & Niedernhofer, 2015; Yousefzadeh et al., 2019). *Ercc1* ^−/∆^ mice, expressing ~5% of the normal complement of ERCC1‐XPF, best model the human disease (Gurkar & Niedernhofer, 2015; Yousefzadeh et al., 2019) and represent the most systemic model of premature onset of age‐related pathology (Harkema, Youssef, & de Bruin, 2016). *Ercc1* ^−/∆^ mice experience the same spontaneous, endogenous DNA damage as WT animals. However, because of their defect in DNA repair, they accumulate the damage faster than WT mice (Robinson et al., 2018; Wang, Clauson, Robbins, Niedernhofer, & Wang, 2012). Like XFE progeroid patients, *Ercc1* ^−/∆^ mice spontaneously develop multiple age‐related morbidities during their 7 month lifespan, including osteoporosis, cardiovascular disease, cataracts, loss of hearing and vision, peripheral neuropathy, hepatic fibrosis, cerebral atrophy with cognitive decline, and intervertebral disk degeneration (Dolle et al., 2011; Gregg et al., 2012; Harkema et al., 2016).

To determine whether *Ercc1* ^−/∆^ mice show an acceleration of the normal aging process and reveal which tissues senesce as a consequence of endogenous DNA damage, we measured numerous markers of senescence in multiple tissues from f_1_ C57BL/6J:FVB/N *Ercc1* ^−/∆^ and compared them with age‐matched WT mice as well as old WT mice. The expression of senescence indices, including the SASP, was increased relative to healthy adult mice to a similar extent in 4‐ to 5‐month‐old *Ercc1* ^−/∆^ and 30‐month‐old WT mice. Likewise, the spectrum of tissues affected was remarkably similar between the progeroid and aged WT mice. These data provide a survey of tissues in which senescent cells accumulate with aging in WT mice. They also provide evidence that *Ercc1* ^−/∆^ mice are undergoing an acceleration of the normal aging process. Our results also contribute evidence that spontaneous, endogenous DNA damage drives senescence in vivo.

## 2 RESULTS

### 2.1 Increased expression of senescence markers in peripheral lymphocytes with aging

**Figure 1:**
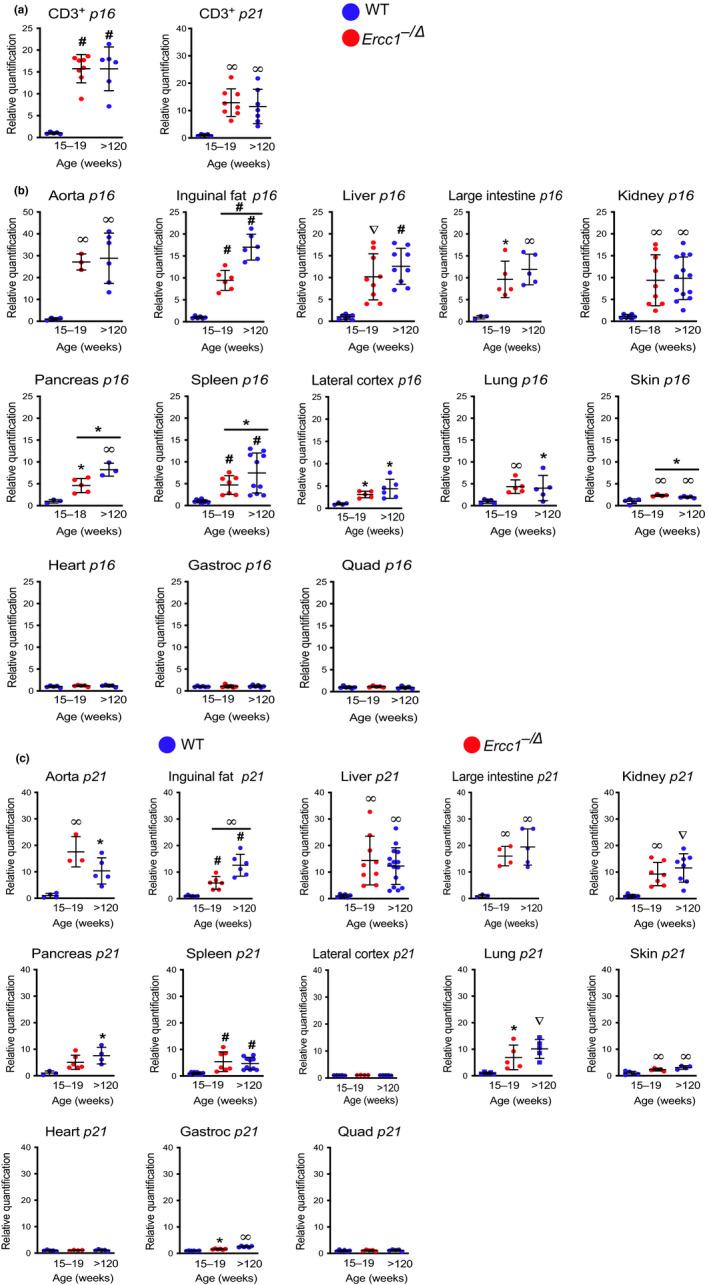
Expression of *p16^Ink4a^* and *p21^Cip1^* mRNA in various murine tissues with aging. (a) Total RNA was isolated from CD3^+^ T lymphocytes purified from the peripheral blood of 15‐ to 19‐week‐old *Ercc1^−/∆^* (red) mice, age‐matched WT controls, and aged WT (>120‐week‐old) mice (blue) (*n* = 5–8 per group) by magnetic bead purification using an anti‐CD3 antibody. Expression of the senescence genes, *p16^Ink4a^* and *p21^Cip1^*, was measured by qPCR. Total RNA was isolated from snap frozen tissues collected from *Ercc1^−/∆^* (red) and WT (blue) mice (*n* = 3–15 mice per group). Expression of (b) *p16^Ink4a^* and (c) *p21^Cip1^* was measured by qPCR using the *∆∆*Ct method and normalized to *Gapdh* expression. Values represent the mean ± *SD*, one‐way ANOVA with Tukey's test. *p < .05, ^∞^ *p* < .01, ^∇^ *p* < .001, ^#^ *p* < .0001

Expression of *p16^Ink4a^* (mRNA) in peripheral blood T cells is a robust marker of human aging (Liu et al., 2009; Rosko et al., 2015). This was recently extended to mice (Liu et al., 2019). As previously, reported for mice, *p16^Ink4a^* expression, as measured by qPCR, was significantly elevated (16X, *p* < .0001) in T cells of 30‐month‐old WT mice relative to 4‐ to 5‐month‐old WT mice in an f_1_ background (50:50 C57BL/6:FVB/n) (Figure 1a). *p21^Cip1^* mRNA levels were similarly elevated in lymphocytes from old WT mice compared with young adults (11X, *p* < .01). Levels of *p16^Ink4a^* and *p21^Cip1^* mRNA in T cells from 4‐ to 5‐month‐old *Ercc1* ^−^ *^/∆^* mice were similarly increased relative to age‐matched WT controls (Figure 1a; 15‐fold; *p* < .0001 and 13‐fold; *p* < .01, respectively). The expression of these senescence markers was indistinguishable between the progeroid and aged WT mice. Of note, error bars were quite large for both the progeroid and old WT mice, reflecting the variability in the aging process, which is well documented (Burd et al., 2013).

### 2.2 Tissue‐specific expression of senescence markers with aging

To determine where senescence occurs in aged WT mice, *p16^Ink4a^* and *p21^Cip1^* mRNA were measured in thirteen tissues and compared with expression in young adult WT mice (Figure 1b–c). The expression of these transcripts was significantly increased in 10 of the 13 tissues analyzed in old relative to young mice. Expression of *p16^Ink4a^* and *p21^Cip1^* was greatest in the aorta of the aged mice (*p16^Ink4a^* ~ 25‐fold; *p* < .01; *p21^Cip1^*~ 10‐fold; *p* < .01). This is consistent with prior reports that endothelial cells readily undergo senescence (Khan et al., 2017; Zhu, Tchkonia, Pirtskhalava, et al., 2015). Skeletal muscle (quadriceps) and heart were the only tissues where both *p16^Ink4a^* and *p21^Cip1^* were not significantly elevated in aged WT mice. Gastrocnemius muscle from aged mice had a modest, but significant increase in *p21^Cip1^* expression, relative to adult WT mice, while the quadriceps did not (Figure 1c). *p21^Cip1^* expression was not significantly increased in the lateral cerebral cortex of aged WT mice, although *p16^Ink4a^* expression was increased twofold to threefold (*p* < .05) (Figure 1b–c). Rank order of *p16^Ink4a^* expression in old WT mice, as measured by qPCR, from highest to lowest was aorta, inguinal fat, liver, large intestine, kidney, pancreas, spleen, brain, lung, and skin in old WT mice (Table 1). For the most part, expression of *p21^Cip1^* followed the same trend.

**Table 1:** Rank order of *p16^Ink4a^* expression in tissues from aged WT and progeroid *Ercc1^−/∆^* mice

Old WT mice	Highest												Lowest
Tissue	Aorta	Inguinal fat	Liver	Large intestine	Kidney	Pancreas	Spleen	Brain	Lung	Skin	Heart	Gastroc	Quad
Avg. fold increase in *p16* expression	28.8	17.0	12.6	11.9	9.9	8.2	7.4	4.4	4.0	1.9	1.1	1.1	1.0
Significance	∞	#	#	∞	∞	∞	#	*	*	∞	ns	ns	ns

*Ercc1^−/∆^* mice	Highest												Lowest
Tissue	Aorta	Liver	Large intestine	Inguinal fat	Kidney	Spleen	Pancreas	Lung	Brain	Skin	Heart	Quad	Gastroc
Avg. fold increase in *p16* expression	27.2	10.2	9.6	9.4	9.4	4.7	4.6	4.4	3.1	2.3	1.2	1.1	1.1
Significance	∞	∇	*	#	∞	#	*	∞	*	∞	ns	ns	ns
Significantly different from old WT	ns	ns	ns	#	ns	*	*	ns	ns	*	ns	ns	ns

* *p* < .05.

^∞^ *p* < .01.

^∇^ *p* < .001.

^#^ *p* < .0001.

The levels of *p16^Ink4a^* mRNA were significantly elevated in the same 10 of 13 tissues analyzed as in aged WT mice (Figure 1b). Notably, of the 13 tissues in which *p16^Ink4a^* mRNA was measured, there were only four where levels differed significantly between old WT and progeroid *Ercc1^−/∆^* mice. *p16^Ink4a^* expression was greater in the inguinal fat (white adipose tissue), pancreas, and spleen of old WT than mutant mice, but lower in the skin. The levels of *p21^Cip1^* mRNA were even more consistent between the progeroid and aged WT mice, with levels of the senescence marker being significantly greater in old WT mice only in the inguinal fat. It is notable that of 14 tissues (13 organs plus lymphocytes), and two senescence markers measured, there was only one example where *Ercc1^−/∆^* mice have a greater signal. This indicates that the *Ercc1^−/∆^* mice are not an exaggerated model of aging, but an accurate one that occurs in a compressed period of time, that is, accelerated aging. Also remarkable, the rank order of senescence marker expression was quite similar in *Ercc1^−^* ^/∆^ mice as in WT mice (Table 1). Of the 26 endpoints measured (two markers in 13 tissues), there were only four tissues (*p16^Ink4a^* in inguinal fat, pancreas, skin, and spleen and *p21^Cip1^* in inguinal fat) where there was a significant difference between *Ercc1^−/∆^* and old WT mice.

### 2.3 Sex differences in senescence marker expression

While the expression of *p16^Ink4a^* and *p21^Cip1^* mRNA was significantly increased in both sexes of *Ercc1^−/∆^* mice, it was significantly greater in males at 4 to 5 months of age than age‐matched females in the liver, kidney, and spleen (Figure S1). Similarly, in 2.5‐year‐old WT mice, expression of *p16^Ink4a^* and *p21^Cip1^* was significantly greater in male mice compared with female (Figure S1). Expression of *p16^Ink4a^* and *p21^Cip1^* mRNA was indistinguishable between old WT mice (30 months) and *Ercc1^−/∆^* mice (4–5‐months‐old) of the same sex with two exceptions. *p21^Cip1^* expression was modestly greater in the liver of *Ercc1^−^* ^/Δ^ male mice compared with old WT males (*p* < .05) and *p16^Ink4a^* was lower in the spleen of *Ercc1^−^* ^/Δ^ male mice than in aged WT male mice (*p* < .01).

The sex disparities in senescence marker expression in old WT mice were also striking. Thus, we asked, at an older age, do females “catch‐up” to males. Indeed, five months later, at 140 weeks of age), the disparity in senescence marker expression between sexes was dramatically diminished (Figure S2). *p16^Ink4a^* mRNA was still significantly lower in the liver and spleen from female mice than from males (*p* < .05) but *p21^Cip1^* mRNA was significantly greater in the spleens of female mice than males (*p* < .05). Collectively, these data provide a map of the location and magnitude of senescent cell accumulation during mouse aging. The data also demonstrate that the tissue specificity, sex disparity, and level of senescent marker expression are very similar between 4‐ to 5‐month‐old *Ercc1^−^* ^/∆^ and 2.5‐year‐old WT mice.

### 2.4 Other measures of senescence

**Figure 2:**
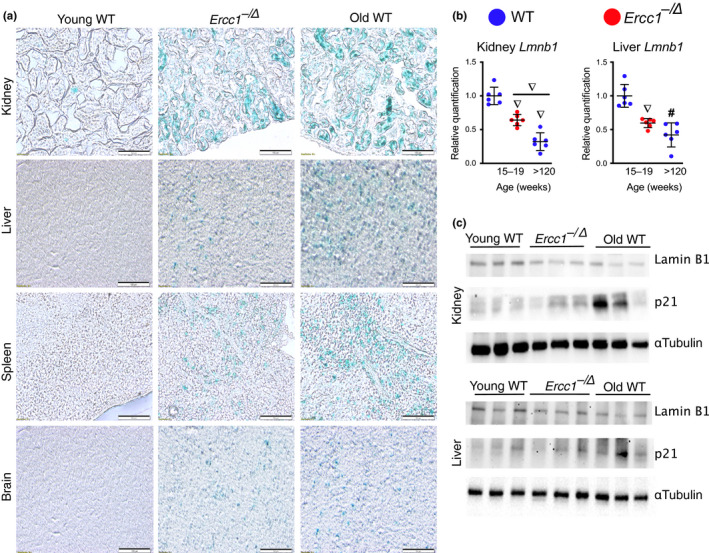
Expression of other senescence markers in WT and progeroid mice. (a) Representative images of fixed frozen samples from 18‐week‐old *Ercc1^−/∆^* mice, age‐matched WT controls, and old WT (>120‐week‐old) mice stained for SA‐βgal activity and imaged at 20X with bright‐field microscopy (scale bar, 100 μm). (b) Total RNA was isolated from snap frozen tissues collected from *Ercc1^−/∆^* (red) and WT (blue) mice (*n* = 6 mice per group). Expression of the senescence marker *LmnB1* was measured by qPCR using the *∆∆*Ct method and normalized to *Gapdh* expression. (c) Lysates from the kidney and liver of 15‐ to 19‐week‐old *Ercc1^−/∆^* mice, age‐matched “young” WT controls and “old” WT (>120‐week‐old) mice were immunoblotted with an anti‐Lamin B1 or ‐p21*^Cip1^* antibodies. α‐Tubulin served as a loading control. Values represent the mean ± *SD*, one‐way ANOVA with Tukey's test. ^∇^ *p* < .001, ^#^ *p* < .0001

To validate the qPCR data using other senescence endpoints, additional measurements of senescence were made. Enhanced lysosomal biogenesis is a common feature of senescent cells (Lee et al., 2006). This is detected by measuring β‐galactosidase activity at pH 6.0 and is termed senescence‐associated β‐galactosidase (SA‐βgal) activity. SA‐βgal staining is one of the most frequently used methods to detect senescence (Dimri et al., 1995; Yousefzadeh et al., 2019). As mammals age, SA‐βgal activity increases in numerous tissues (Dimri et al., 1995; Melk et al., 2003; Pendergrass et al., 1999). Consistent with this, SA‐βgal^+^ cells were present in the brain, kidney, liver, and spleen of *Ercc1^−/∆^* and aged WT mice, but not in young WT animals (Figure 2a). Senescence was further confirmed by measuring expression of *Lmnb1*, whose gene product, Lamin B1, is responsible for maintaining nuclear architecture and is lost during senescence (Freund, Laberge, Demaria, & Campisi, 2012). *Lmnb1* expression was significantly diminished in the kidney and liver of *Ercc1^−/∆^* and old WT mice, relative to adult WT mice (Figure 2b). This was confirmed by immunoblotting for Lamin B1 protein, which was decreased in tissues of aged WT and progeroid mice (Figure 2c). This decrease in Lamin B1 was concomitant with an increase in p21*^Cip1^* protein levels (Figure 2c), which corroborates the qPCR data shown in Figure 1c.

**Figure 3:**
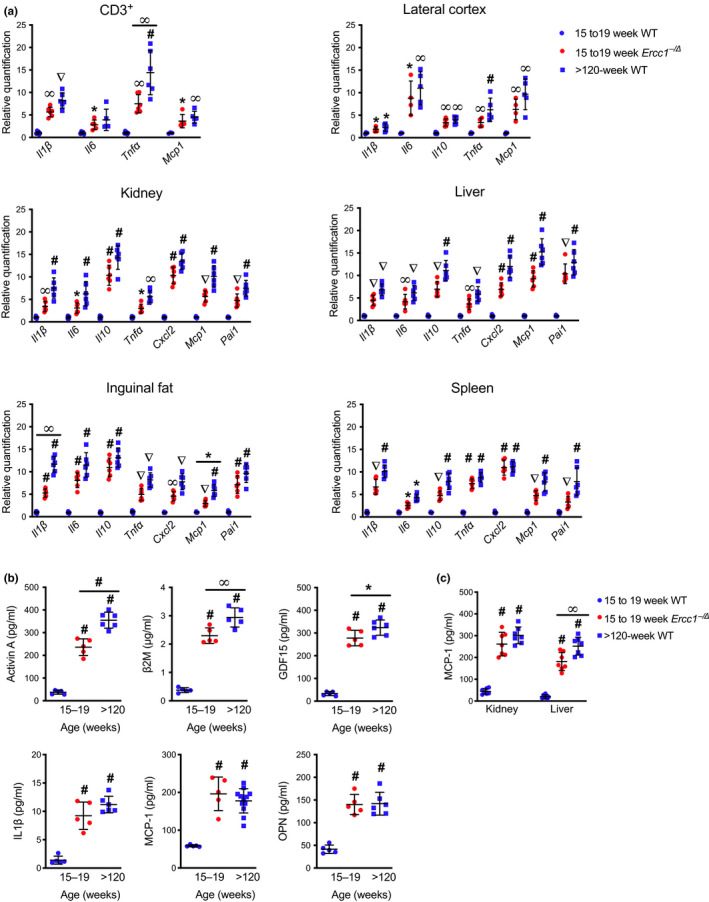
Expression of senescence‐associated secretory phenotype genes in various murine tissues with aging. (a) Total RNA was isolated from CD3^+^ T lymphocytes purified from peripheral blood or from snap frozen tissues isolated from 15‐ to 19‐week‐old *Ercc1^−/∆^* mice, age‐matched WT controls, and “old” WT (>120‐week‐old) mice (*n* = 3–7 per group). Expression of senescence‐associated secretory phenotype (SASP) genes was measured by qPCR using the *∆∆*Ct method and normalized to *Gapdh* expression. (b) Circulating levels of SASP and pro‐geronic factors were quantified in the serum of mice by ELISA (*n* = 5–19 mice per group). (c) Tissue levels of the SASP factor MCP‐1 were quantified by ELISA (*n* = 5–12 mice per group). Values represent the mean ± *SD*, one‐way ANOVA with Tukey's test. *p < .05, ^∞^ *p* < .01, ^∇^ *p* < .001, ^#^ *p* < .0001

A subset of SASP factors were measured by qPCR and ELISA. *Il1β*, *Il6, Il10*, *Tnfα Cxcl2, Mcp1, and Pai1* were measured in five tissues and peripheral T cells using qRT‐PCR (Figure 3a). Expression of all six of these cytokines and chemokines and *Pai1* were significantly elevated in brain, liver, kidney, white adipose tissue, and spleen of aged mice relative to young adult WT mice, as well as in peripheral blood T cells. Expression of the SASP factors was more variable than expression of the cell cycle regulators measured in Figure 1. Generally speaking, SASP factor expression was equal or greater in tissues from aged WT mice compared with the 4‐ to 5‐month‐old *Ercc1^−/∆^* mice.

Another common feature of cellular senescence is the age‐related increase in circulating SASP and pro‐geronic factors (Coppe et al., 2010; Tchkonia et al., 2013). Serum levels of SASP factors Activin A, IL‐1β, GDF15, MCP‐1, and osteopontin (OPN) were elevated in aged WT and 4‐ to 5‐month‐old *Ercc1^−/∆^* mice compared with young WT controls (Figure 3b). β‐2 microglobulin (β2M) was elevated in the serum of aged WT mice, consistent with prior findings (Smith et al., 2015). Circulating β2M was also significantly higher in *Ercc1^−/∆^* mice relative to their littermate controls. Previously, MCP‐1, a chemokine responsible for monocyte recruitment, was shown to increase with chronological and biological age (Yousefzadeh, Schafer, et al., 2018). Circulating MCP‐1 concentrations were significantly higher in *Ercc1^−/∆^* mice compared with their age‐matched, WT counterparts (Figure 3b). Serum levels of MCP‐1 in progeroid mice were comparable to those of old WT mice. Furthermore, MCP‐1 abundance in the liver and kidney of *Ercc1^−/∆^* and aged WT mice was significantly higher than in young WT animals (Figure 3c). In the liver, MCP‐1 protein was greater in old WT mice than in the *Ercc1^−/∆^* mice. These data demonstrate that the profile of changes in SASP marker expression and abundance of circulating SASP factors is consistent between aged wild‐type and *Ercc1^−/∆^* progeroid mice.

### 2.5 Age at onset of senescence in *Ercc1^−/∆^* mice

**Figure 4:**
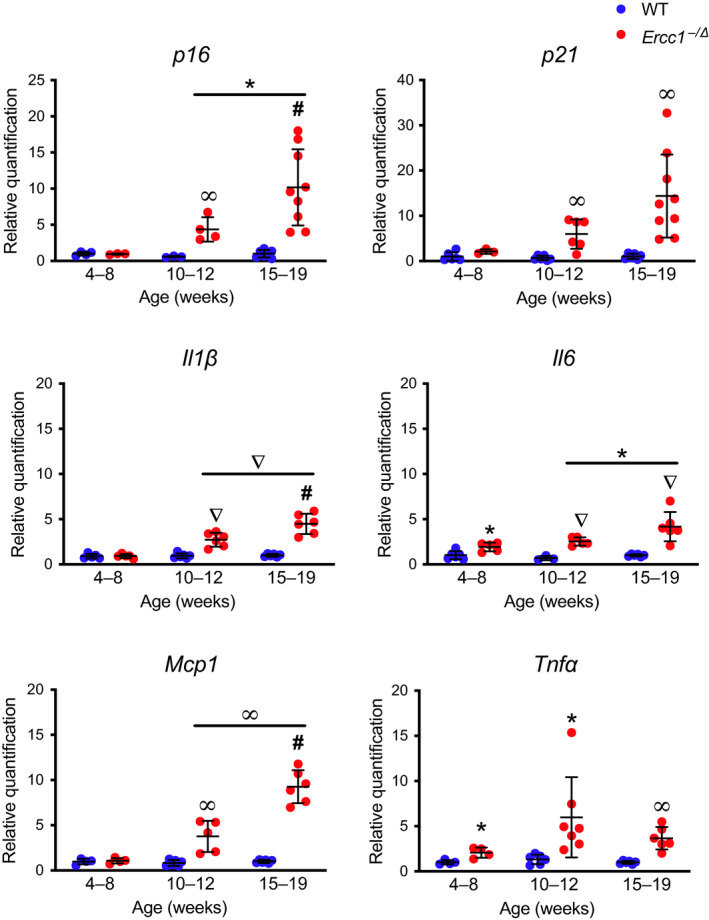
Progressive increase in expression of senescence markers in *Ercc1^−/∆^* mice. Total RNA was isolated from snap frozen liver collected from *Ercc1^−/∆^* (red) and WT (blue) mice (*n* = 4–9 mice per group). Senescence (*p16^Ink4a^* and *p21^Cip1^*) and SASP factor (*Il1β*, *Il6, Mcp1,* and *Tnfα*) expression were measured by qPCR. Relative expression level was quantified using the *∆∆*Ct method and normalized to *Gapdh* expression. Values represent the mean ± *SD*, two‐way ANOVA, and Tukey's test. *p < .05, ^∞^ *p* < .01, ^∇^ *p* < .001, ^#^ *p* < .0001

If *Ercc1^−/∆^* mice experience accelerated physiological aging, then one expects that the onset of senescence is postnatal and that senescent cells accumulate over time. To test this, we measured the expression of *p16^Ink4a^*, *p21^Cip1^*, *Il1β, Il6, Tnfα* and *Mcp1* in the liver of *Ercc1^−/∆^* mice and WT littermates at three ages across the lifespan of the progeroid animals (Figure 4). Expression of *p16^Ink4a^* and *p21^Cip1^* mRNA in young *Ercc1^−/∆^* mice (4–8 weeks) was not significantly different from WT controls, as measured by qPCR. However, by 10–12 weeks of age there was a significant increase in the expression of *p16^Ink4a^* and *p21^Cip1^* in *Ercc1^−/∆^* mice compared with controls. The levels of *p16^Ink4a^* further increased significantly by 15–19 weeks of age in the mutant animals. Expression of SASP factors was also significantly elevated in progeroid mice compared with controls at the two older ages (Figure 4). Expression of *Il6* was significantly higher in *Ercc1^−/∆^* mouse liver by 4–8 weeks of age and increased as the animals aged. These data demonstrate that senescence is not congenital in *Ercc1^−/∆^* mice and that levels of senescence markers increase as the animals age. This is consistent with a prior report that telomere‐associated foci (TAFs, a senescent cell marker)‐positive cells and foci per cell increase with age in two tissues of a mutant mouse strain with progeroid features (Jurk et al., 2014). We previously reported that spontaneous oxidative DNA adducts accumulate in liver and kidney of *Ercc1^−/∆^* mice as they age (Wang et al., 2012). This correlates with the time‐dependent increase in senescence reported here, suggesting a causal relationship.

### 2.6 Detection of senescence in vivo

**Figure 5:**
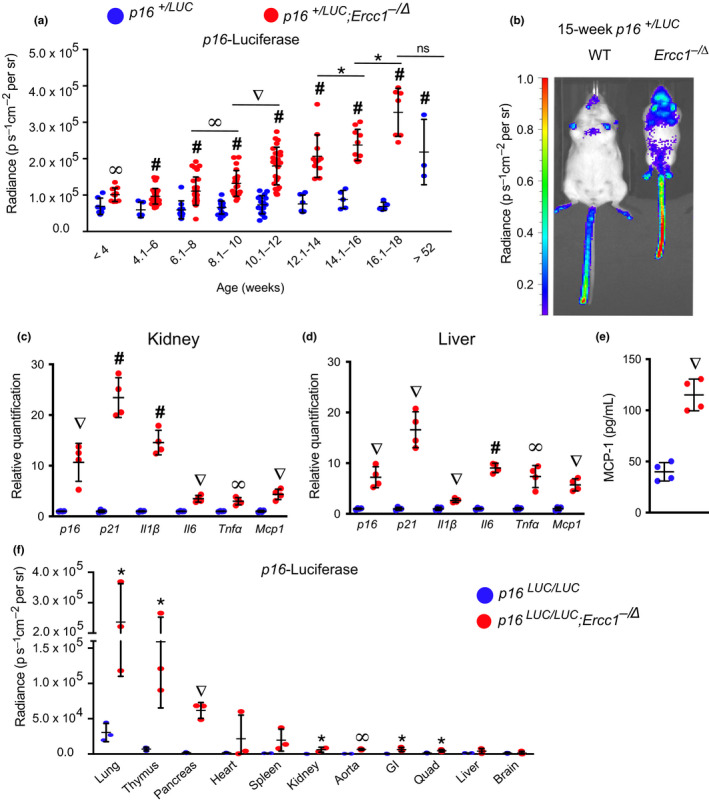
Longitudinal quantification of *p16^Ink4a^* expression. (a) Total body luciferase activity in *p16^+/LUC^;Ercc1^−^* ^/Δ^ (red) and *p16^+/LUC^* (blue) mice with increasing age (*n* = 9–30 mice per group). (b) Representative images of in vivo luciferase imaging in a 15‐week‐old *p16^+/LUC^;Ercc1^−^* ^/Δ^ mouse and a *p16^+/LUC^* littermate. Total RNA was isolated from (c) kidney and (d) liver collected from 11‐week‐old *p16^+/LUC^;Ercc1^−^* ^/Δ^ mice (red) and *p16^+/LUC^* littermates (blue) (*n* = 4 mice per group). Senescence (*p16^Ink4a^* and *p21^Cip1^*) and SASP factor (*Il1β*, *Il6, Mcp1,* and *Tnfα*) expression was measured by qPCR. Relative expression level was quantified using the *∆∆*Ct method and normalized to *Gapdh* expression. (e) Serum levels of the SASP factor MCP‐1 were quantified in 11‐week‐old mice by ELISA. (f) Total luciferase activity in tissues harvested from 17‐week‐old *p16^LUC/LUC^;Ercc1^−^* ^/Δ^ (red) and *p16^LUC/LUC^* (blue) mice (*n* = 3 per genotype). Values represent the mean ± *SD*, unpaired two‐tailed Student's *t* test and two‐way ANOVA with Tukey's test. *p < .05, ^∞^ *p* < .01, ^∇^ *p* < .001, ^#^ *p* < .0001

Longitudinal measurements of senescence in mice were challenging until the transgenic *p16^LUC^* reporter mouse was generated (Burd et al., 2013). The *p16^LUC^* murine strain, which carries a luciferase reporter gene knocked into the native *Cdkn2a* locus, has been used to quantify *p16^Ink4a^* transcription in vivo in WT mice with age (Burd et al., 2013). Luminescence signal is detectable at 4 months of age and steadily rises sevenfold by 20 months of age. For comparison, we used the same transgenic reporter allele to measure *p16* *^Ink4a^* expression longitudinally in an *Ercc1^−/∆^* background. Luciferase signal was already significantly elevated in *p16^+/LUC^;Ercc1^−/∆^* compared with control *p16^+/LUC^* animals at 4 weeks (Figure 5a–b). The luciferase signal increased in *p16^+/LUC^;Ercc1^−/∆^* animals in an age‐dependent manner. It is notable that at no time point did the *p16^+/LUC^;Ercc1^−/∆^* mice have greater luminescence signal than ~1‐year‐old *p16^+/LUC^* control mice (Figure 5a). This comparison also indicates that *Ercc1^−/∆^* and WT mice have roughly equivalent p16‐luciferase signals approximately halfway through their lifespan.

Expression of *p16^Ink4a^* and other senescence markers was confirmed in two tissues of *p16^+/LUC^;Ercc1^−/∆^* reporter mice at 11 weeks of age. (Figure 5c–d). MCP‐1 protein was elevated in serum from the 11‐week‐old reporter mice (Figure 5e), which was consistent with earlier data (Figure 3b). Finally, ex vivo imaging of organs from 17‐week‐old *p16^LUC/LUC^;Ercc1^−/∆^* mice revealed a significant increase in luciferase signal in many tissues (aorta, GI, kidney, lung, muscle, pancreas, and thymus, Figure 5f). Senescence‐driven luminescence was elevated at 4–8 weeks of age (Figure 5a) before a significant increase in *p16^Ink4a^* mRNA was detectable by qRT‐PCR (Figure 4). This discrepancy could indicate a difference in the sensitivity of the two methods, luciferase activity being an enzymatic assay that can amplify signal. Nevertheless, the data demonstrate that senescent cells accumulate over time in *Ercc1^−^* ^/∆^ mice, as occurs with aging in WT mice (Burd et al., 2013).

## 3 DISCUSSION

In this study, we provide a comprehensive measure of senescence in aged WT mice. Senescence was quantified in multiple tissues, using numerous methods and numerous molecular endpoints, and we compared measures with that of young adult WT mice. We used this as a benchmark to determine whether *Ercc1^−/∆^* mice accumulate senescent cells in physiologically relevant tissues. As measured by qRT‐PCR and *p16^LUC^* signal, levels of *p16^Ink4a^* were significantly increased in aged WT mice compared with younger adult mice, as expected (Figures 1 and 5), *p16^Ink4a^* and *p21^Cip1^* expression are found in peripheral T cells and numerous tissues (10 of 14 total tested) with the exception of heart and skeletal muscles. The differences in senescent cell burden in tissues could be reflective of different levels of genotoxic stress and/or different responses to that stress (e.g., selection of cell fate decisions: senescence or apoptosis). Near complete concordance was found between the expression of senescence markers in aged WT (2.5 years) and progeroid *Ercc1^−^* ^/∆^ (4–5 month) mice, in terms of tissue specificity and expression levels (Figure 1, Table 1). These findings were confirmed at the protein level when possible by immunoblotting for p21*^Cip1^* and Lamin B1 (Figure 2c). The systemic burden of senescent cells was equivalent in middle‐aged *Ercc1^−^* ^/∆^ and WT mice, although the strains have vastly different lifespans (Figure 5). This supports the notion that senescent cell burden correlates with organismal health and may prove to be useful in predicting health span, or the remaining fraction of life that is disease‐free. The data also support the conclusion that *Ercc1^−^* ^/∆^ mice spontaneously develop senescent cells in the same tissues and at similar levels as WT mice, albeit more rapidly, supporting the notion that these animals represent a model of accelerated aging.

Interestingly, the highest levels of *p16^Ink4a^* and *p21^Cip1^* were detected in the aorta of *Ercc1^−/∆^* and aged WT mice (Figure 1b–c, Table 1), suggesting that this tissue may be hypersensitive to endogenous DNA damage that induces senescence and/or vulnerable to circulating SASP factors. Expression of both senescence and SASP markers was significantly elevated in peripheral T cells of *Ercc1^−/∆^* and old WT mice (Figures 1a and 3a). Likewise, senescence marker expression was elevated in the skin of both aged wild‐type and progeroid mice. Peripheral T cells and skin, which are readily accessible organs, or the aorta, with its large dynamic range of senescence marker expression, may prove useful for measuring the response to senolytic interventions in mice (Yousefzadeh, Zhu, et al., 2018). We did not detect increased expression of *p16^Ink4a^* and *p21^Cip1^* in skeletal muscle or the heart of aged WT or young adult *Ercc1^−^* ^/∆^ mice (Figure 1b–c). This is in contrast to the *BubR1^H/H^* mouse model, which also has progeroid features, that shows increased *p16^Ink4a^* expression in skeletal muscle, inguinal adipose tissue, and eye, but not liver or heart (Baker et al., 2004). SASP marker expression was also comparable in extent and abundance in multiple tissues of old WT and *Ercc1^−^* ^/∆^ mice. Serum analysis revealed that the pattern of age‐associated increase of SASP factors in normal aging is also reflected in mice experiencing accelerated aging (Figure 3b).

Senescence and SASP marker expression were monitored over the lifespan of *Ercc1^−^* ^/∆^ mice (Figures 4 and 5). After reaching adulthood, the senescence and SASP markers were significantly increased in multiple tissues and by multiple measures, and continued to rise as the animals aged (Figures 4 and 5). This correlates with the onset and progression of age‐related pathologies in multiple tissues of the *Ercc1^−^* ^/∆^ model (Dolle et al., 2011; Gregg et al., 2012; Gurkar & Niedernhofer, 2015; Harkema et al., 2016; Yousefzadeh et al., 2019; Yousefzadeh, Zhu, et al., 2018), providing further evidence that senescent cells play a causal role in numerous age‐related pathologies (Baker et al., 2016).

Expression of the SASP factors is significantly higher in 10‐ to 12‐week‐old *Ercc1^−^* ^/∆^ mice as compared to WT littermates, which appears to coincide with a detectable rise in *p16^Ink4a^* and *p21^Cip1^* mRNA. However, we observed elevated *Il6* expression in *Ercc1^−^* ^/∆^ mice by 4–8 weeks (Figure 4). This antecedent expression of cytokines could be due to DNA damage‐induced expression of NF‐κB‐dependent pro‐inflammatory mediators like TNF*α* and IL‐6 (Nakad & Schumacher, 2016) prior to the induction of senescence. Previously, we demonstrated that NF‐κB is activated in tissues from *Ercc1^−^* ^/∆^ mice and that inhibition of NF‐κB, through genetic depletion of the *p65* subunit, delayed the onset of cellular senescence and aging in *Ercc1^−^* ^/∆^ animals (Tilstra et al., 2012).

Peripheral blood CD3^+^ cells had elevated levels of SASP factors that could contribute to secondary senescence and promote systemic aging (Figure 3a) (Coppe et al., 2010; Sharpless & Sherr, 2015). Given that *p16^Ink4a^* mRNA, as well as chemokines and cytokines, increase with age in peripheral blood cells (Liu et al., 2009; Sharpless & Sherr, 2015), measuring this senescence “signature” in blood samples could be a rapid way to assess biological age, as opposed to chronological age (Yousefzadeh, Zhu, et al., 2018). Notably *Il1β, Il6, Tnfα* and *Mcp1* expression are elevated in peripheral blood mononuclear cells from progeroid *Ercc1^−^* ^/∆^ mice (Figure 3a), further strengthening the parallels with normal aging and the utility of this strain for testing senolytics.

Interestingly, senescence appeared to be higher in *Ercc1^−^* ^/∆^ males at 4–5 months of age than age‐matched females. Similarly, in aged WT mice, expression of *p16^Ink4a^* and *p21^Cip1^* was significantly greater in male compared with female mice at ~120 weeks of aging. However, near the end of life in WT mice (140 weeks of age), the difference in expression of senescence markers between male and female mice was not significant. This suggests that near the end of life, there is a greater increase in the rate of senescence in female mice. These results also suggest that the extent of senescence could explain differences in severity of different types of age‐related diseases between male and female mice.

Taken together, our data demonstrate that *Ercc1^−^* ^/∆^ mice share a pattern of cellular senescence that is consistent and commensurate with that observed in aged WT mice. The DNA repair‐deficient *Ercc1^−^* ^/∆^ mice accumulate endogenous DNA damage faster than WT mice (Robinson, 2018; Wang et al., 2012), and we now show that this corresponds with the accelerated accumulation of senescent cells. This supports the conclusions that spontaneous, endogenous DNA damage drives cellular senescence in vivo with natural aging and that the *Ercc1^−^* ^/∆^ mice truly age in an accelerated manner. *Ercc1^−^* ^/∆^ mice therefore offer a rapid and cost‐effective model for the evaluation of senotherapeutics.

## 4 METHODS

### 4.1 Animals

All animal studies were conducted in compliance with the U.S. Department of Health and Human Services Guide for the Care and Use of Laboratory Animals and were approved by the Scripps Florida and University of Minnesota Institutional Animal Care and Use Committees. *Ercc1^−^* ^/∆^ mice were bred as previously described (Ahmad et al., 2008). *p16*‐luciferase reporter mice were obtained from Ohio State University (Burd et al., 2013) and bred to create albino C57Bl/6 p16^+/LUC^;*Ercc1* ^±^ and FVB/n p16^+/^ *^LUC^*;*Ercc1* ^+/∆^ mice. These mice were further crossed to create f_1_ p16^+/^ *^LUC^*;*Ercc1^−^* ^/^ *^Δ^* mice with white fur for imaging. All animals were genotyped from an ear punch by TransnetYX.

### 4.2 RNA isolation and qPCR

Analysis of senescence‐associated mRNAs in tissues was performed as described in Yousefzadeh et al. (2019). Tissues were harvested from euthanized animals and snap frozen in liquid nitrogen. Tissues were homogenized using FastPrep‐24 homogenizer (MP Biomedicals), and total RNA was isolated by TRIzol extraction according to manufacturer's specifications (Thermo Fisher). Total RNA was quantified using a NanoDrop spectrophotometer (Thermo Fisher) and 1 μg of total RNA used to generate cDNA via the Transcriptor First Strand cDNA Synthesis Kit (Roche) according to the manufacturer's specifications. Gene expression changes were quantified by qPCRs using 20 μl reaction volumes and a StepOne thermocycler (Thermo Fisher) with input of 50 ng total RNA per reaction (except *p16^Ink4a^*, 100 ng total RNA). For each sample, reactions were performed in duplicate (*n* = 3–15 individual tissue per group). Data was analyzed by ΔΔCt method and the expression was normalized to *Gapdh*. Primer sequences were as follows: *Cdkn1a* (*p21^Cip1^*) Rev 5′‐CGGTCCCGTGGACAGTGAGCAG‐3'; *Cdkn2a* (*p16^Ink4a^*) Fwd 5′‐ CCCAACGCCCCGAACT*‐3*′*, Cdkn2a* (*p16^Ink4a^*) Rev 5′‐GCAGAAGAGCTGCTACGTGAA‐3′; *Cxcl2* Fwd 5′‐CCTGGTTCAGAAAATCATCCA‐3′, *Cxcl2* Rev 5′‐CTTCCGTTGAGGGACAGC‐3′; *Gapdh* Fwd 5′‐AAGGTCATCCCAGAGCTGAA‐3′, *Gapdh* Rev 5′‐CTGCTTCACCACCTTCTTGA‐3′; *Il1β* Fwd 5′‐ CACAGCAGCACATCAACAAG‐3′, *Il1β* Rev 5′‐GTGCTCATGTCCTCATCCTG‐3′; *Il6* Fwd 5′‐CTGGGAAATCGTGGAAT‐3′, *Il6* Rev 5′‐CCAGTTTGGTAGCATCCATC‐3′; *Il10* Fwd 5′‐ATAACTGCACCCACTTCCCA‐3′, *Il10* Rev 5′‐GGGCATCACTTCTACCAGGT‐3′; *Lmnb1* Fwd 5'‐GGGAAGTTTATTCGCTTGAAGA‐3', *Lmnb1* Rev 5'‐ATCTCCCAGCCTCCCATT‐3'; *Mcp1* Fwd 5'‐GCATCCACGTGTTGGCTCA‐3', *Mcp1* Rev 5'‐CTCCAGCCTACTCATTGGGATCA‐3'; *Pai1* Fwd 5'‐GACACCCTCAGCATGTTCATC‐3', *Pai1* Rev 5'‐AGGGTTGCACTAAACATGTCAG‐3'; *Tnf‐α* Fwd 5′‐ATGAGAAGTTCCCAAATGGC‐3′, *Tnf‐α* Rev 5′‐CTCCACTTGGTGGTTTGCTA‐3′.

### 4.3 Isolation of peripheral blood CD3 + T lymphocytes

Analysis of senescence‐associated mRNAs in CD3 + T cells was performed as described in (Yousefzadeh et al., 2019). Blood was obtained from mice (*n* = 5–8 per group) by cardiac puncture, immediately placed into 1/10th volume of 0.5 m EDTA and gently mixed to prevent coagulation. Samples were centrifuged at 300 *g* for 10 min in a tabletop centrifuge. Supernatant was discarded, and the cell pellet was suspended in 1 ml ACK buffer (150 mm NH_4_Cl, 10 mm KHCO_3_, 0.1 mm Na_2_EDTA, pH 7.4) and then incubated at room temperature for 10 min to lyse the red blood cells. Cells were spun down and ACK lysis repeated for a second time. Cells were then spun down, washed in PBS, and resuspended in PBS containing 0.5% FBS and 2 mm EDTA. 50 µl anti‐CD3‐Biotin conjugate (Miltenyi Biotec) was added to the cell suspension solution and incubated for 30 min on ice. Cells were centrifuged at 300 *g* for 10 min and washed twice in resuspension buffer. The cell pellet was then resuspended in 500 µl of resuspension buffer and 100 µl of anti‐biotin microbeads added before a 15‐min incubation on ice. Cells were washed twice and then resuspend in 500 µl of resuspension buffer and applied to a MACS column attached to a magnet. Cells were washed with 3X the column volume of buffer before elution. Cells were centrifuged and RNA isolation conducted using a RNeasy kit (Qiagen) according to manufacturer's specifications. qPCR analysis of senescence markers was performed as indicated above.

### 4.4 Analysis of SASP and pro‐geronic factors

Circulating and tissue levels of Activin A (R&D Systems), β‐2 microglobulin (β2M, Abcam), GDF15 (R&D Systems), IL‐1β (Abcam), MCP‐1 (RayBiotech), and Osteopontin (OPN, R&D Systems) were measured by ELISA using a Varioskan plate reader (Thermo Fisher) according to manufacturer's specifications.

### 4.5 Immunoblotting

Snap frozen livers from mice were incubated in RIPA buffer with Complete Mini protease inhibitor (Roche) on ice for 30 min (Thermo Fisher) after being homogenized with a FastPrep‐24 homogenizer. Samples were centrifuged at 17,000 *g* for 15 min at 4°C. Supernatant was resuspended in 2X SDS loading buffer and 50 µg of total protein run on a 4%–15% SDS‐PAGE gel before being transferred to a nitrocellulose membrane. Membranes were blocked for 1 hr in 10% milk TBS‐T solution at room temperature before incubation in anti‐Lamin B1 (Abcam, catalog# ab16048, 1:1,000), anti‐p21 (Santa Cruz Biotechnology, catalog# sc‐6246, 1:400) or anti‐*α*Tubulin (Abcam, catalog# ab6046, 1:2000) antibody at 4°C overnight. After washing, samples were incubated in goat anti‐rabbit or anti‐mouse HRP secondary antibody (Sigma, catalog # A0168 and A0545, 1:2,500) in 5% milk TBS‐T solution for 3 hr before washing and visualization with ECL (Thermo Fisher).

### 4.6 Senescence‐associated β‐galactosidase (SA‐βgal) staining

SA‐βgal staining was performed as described in (Yousefzadeh et al., 2019). Fresh tissues from 15‐ to 19‐week‐old *Ercc1^−^* ^/^ *^Δ^* and WT littermate controls (labeled young) and >120‐week‐old WT mice (*n* = 3–4) were fixed in 10% neutral buffered formalin (NBF) for 4 hr and then transferred to 30% sucrose overnight. Tissues were then embedded in cryo‐embedding media (OCT) and cryosectioned at 5 μm. SA‐βgal staining (pH 5.8–6.0) of tissue sections was performed at 37°C for 16–24 hr in SA‐βgal staining solution. Images were captured using bright‐field microscopy at 20X magnification.

### 4.7 IVIS in vivo imaging detection of luciferase activity

Isoflurane‐anesthetized mice were injected intraperitoneally with 10 μL per gram body weight D‐luciferin substrate (Caliper Life Sciences; 15 mg/ml in PBS) and were imaged using an IVIS Lumina (Caliper Life Sciences) as previously described (Burd et al., 2013; Yousefzadeh et al., 2019).

## CONFLICTS OF INTEREST

None declared.

## AUTHOR CONTRIBUTIONS

MJY, PDR, and LJN designed the experiments. MJY, JZ, AUG, MPB, CAM, MC, JIK, and CB measured senescence in postmortem tissues. MJY, SJM, LA, and EAW measured senescence in vivo. CEB contributed *p16^LUC^* mice and to data analysis. MJY, LJN, CEB, and PDR helped prepare the manuscript.

## Data Availability

The data that support the findings of this study are available from the corresponding author upon reasonable request.
