# Supplementary material for: Tissue specificity of senescent cell accumulation during physiologic and accelerated aging of mice
Source: Aging Cell. 2020 Jan 25;19(3):e13094. doi: 10.1111/acel.13094 (PMC7059165; doi:10.1111/acel.13094)
Supplement: Supplementary file 1 [file ACEL-19-e13094-s002.docx]

**Figure S1. Sex-based differences in senescence marker expression with aging.** Total RNA was isolated from snap frozen tissues collected from 15-19-week-old male and female *Ercc1^-/∆^* (red) mice (n=3-5 mice per group). Expression of the senescence markers, (A) *p16^Ink4a^* and (B) *p21^Cip1^*, was measured by qPCR using the *∆∆*Ct method and normalized to *Gapdh* expression. Senescence was normalized to age-matched WT controls (noted by the black dashed line). These are the same data as presented in Figure 1, but separated by gender. Values represent the mean ± SD, two-way ANOVA and Tukey’s test. *p<0.05, ^∞^p<0.01, ^∇^p<0.001, ^#^p<0.0001.

**Figure S2. Sex-based differences in senescence marker expression in aged WT mice.** Total RNA was isolated from snap frozen tissues collected from 140-week-old male and female WT mice (n=3-4 per group). Expression of the senescence markers (A) *p16^Ink4a^* and (B) *p21^Cip1^* was measured by qPCR using the *∆∆*Ct method and normalized to *Gapdh* expression. Senescence was normalized to 15-19-week-old WT controls (noted by the black dashed line). Values represent the mean ± SD, two-way ANOVA and Tukey’s test. *p<0.05, ^∞^p<0.01, ^∇^p<0.001, ^#^p<0.0001.
