# Supplementary figures and images for: Tissue specificity of senescent cell accumulation during physiologic and accelerated aging of mice
Source: Aging Cell. 2020 Jan 25;19(3):e13094. doi: 10.1111/acel.13094 (PMC7059165; doi:10.1111/acel.13094)

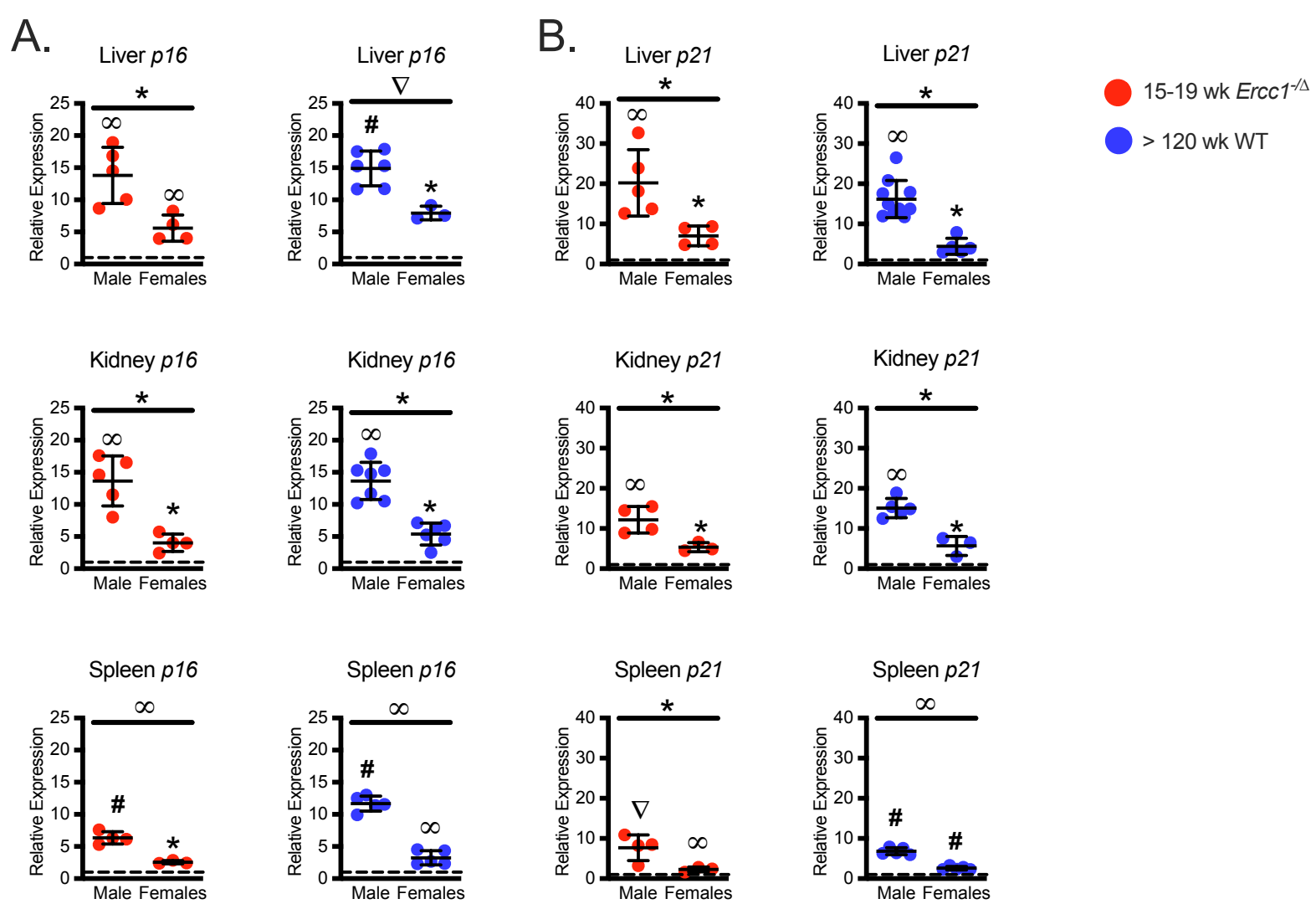

Supplemental Figure 1

A.

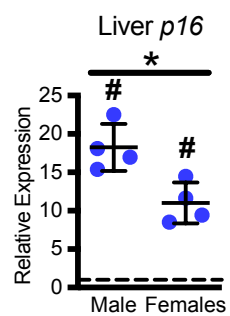

B.

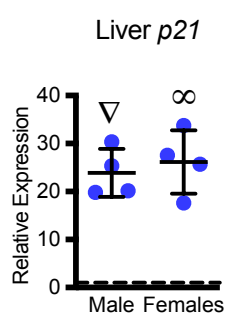

● > 140 wk WT

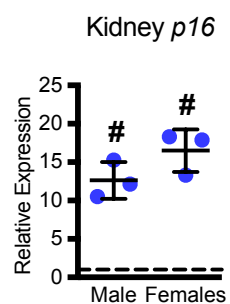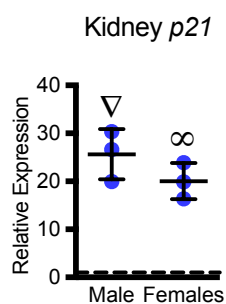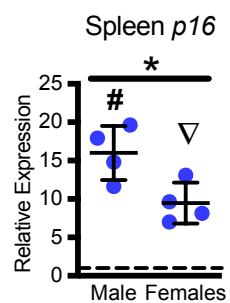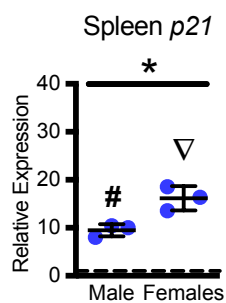

Supplement: Supplementary file 2 [file ACEL-19-e13094-s001.pdf]
